# Hidden diversity in *Prochilodus nigricans*: A new genetic lineage within the Tapajós River basin

**DOI:** 10.1371/journal.pone.0237916

**Published:** 2020-08-25

**Authors:** Ueslei Lopes, Pedro M. Galetti, Patricia Domingues de Freitas

**Affiliations:** 1 Departamento de Genética e Evolução, Universidade Federal de São Carlos, São Carlos, SP, Brazil; 2 Centro de Ciências da Natureza, Campus Lagoa do Sino, Universidade Federal de São Carlos, Buri, SP, Brazil; Natural History Museum of London, UNITED KINGDOM

## Abstract

Highly spread through the Amazon River basin, *Prochilodus nigricans* have had its taxonomic validity recently questioned, when genetic differences between Western and Eastern Amazon populations from the Brazilian shield were detected. This area has been seeing as a region of high ichthyofaunal diversity and endemism, in which the hybrid origin of the Tapajós River basin has been raised. In this paper, we report a new molecular lineage within *P*. *nigricans* of Tapajós River, highlighting this region still hides taxonomically significant diversity. Haplotype networks were reconstructed using the mitochondrial COI and ATP6/8 markers, which were also used to calculate genetic distances among clusters. We additionally conducted a delimiting species approach by employing a Generalized Mixed Yule-Coalescent model (GMYC) with COI sequences produced here, and previous ones published for individuals sampled across the Amazon River basin. In addition to the genetic differentiation within *P*. *nigricans*, our findings favor the hypothesis of hybrid origin of the Tapajós River basin and reaffirm the importance of studies aiming to investigate hidden diversity to address taxonomic and biogeographic issues, that certainly benefit better biodiversity conservation actions.

## 1. Introduction

Freshwater ecosystems are exposed to great human-promoted impacts and transformations [1], making studies focusing on the discovery and comprehension of the extent biodiversity crucial for their conservation [2,3]. With over 5,160 freshwater fish species described in the South American rivers, this region harbors one-third of fish species of the entire planet, and the expectation is that this number is 42% higher [4]. Under this perspective, the Amazon River basin occupies a remarkable position, since its large extension is home for a huge diversity of fish species [5], many of which remain unknown.

Among the fish diversity from the Amazon basin, *Prochilodus nigricans* (Prochilodontidae, Characiformes) is one of the three species of the genus found in the Amazon River basin and presents the largest geographic distribution through the drainage in comparison with its congeners [6]. Known as Black prochilodus or curimbatá, *P*. *nigricans* is an abundant species that initiates spawning migration as soon as the flooding season starts [7,8]. With a detritivorous diet, this fish plays an essential functional role in ecosystems by modulating the fluxes of energy and nutrients [9–12]. *P*. *nigricans* also assumes an important economic and social role in Brazil, since it is one of the dominant species in local fisheries and highly used by the riverine community for subsistence [13,14].

Recently, studies on molecular phylogeny have questioned the monophyly of *P*. *nigricans* pointing out the existence of two mitochondrial lineages in the Amazon River basin [15,16]. A lineage includes specimens of *P*. *nigricans* from lowlands of Western Amazon and its mainstream, while a second one is considered a complex of species that includes what is described as *P*. *nigricans* from uplands of the Eastern Amazon (Araguaia River, Upper and Middle Tapajós River), *P*. *britskii* from Apiacás (Upper Tapajós River), *P*. *brevis* from northeastern Brazil (Ceará and Rio Grande do Norte states), *P*. *lacustris* from Parnaíba River, and *P*. *rubrotaeniatus* from the Upper Orinoco and Upper Essequibo River basins [15,16]. Namely, within this complex lineage two taxonomic units, *P*. *britskii* and the *P*. *nigricans* Eastern Amazon group, were also found in the Tapajós River basin.

The Amazon biogeography is quite complex, and this is particularly evident in the Tapajós River basin. Distinct cladistic approaches and a broad sampling across the Amazon showed an intricate history for this hydrographic system, in which the Tapajós River basin was depicted as non-monophyletic, showing a high degree of historical hybridism [17]. In this scenario, the occurrence of new taxonomic units into the Tapajós River basin can be expected. Considering the Brazilian Shield is an underestimated region of high ichthyofaunal diversity and endemism [18], and the previously reported *Prochilodus* phylogeny [15,16] included few individuals from Tapajós River, this basin still requires a more extensive sampling.

In this sense, and taking into account the importance of DNA-based approaches for delimiting species [5,19–22], we analyzed *P*. *nigricans* throughout the Tapajós River basin to investigate if this hydrographic system still hides taxonomically significant diversity for this important fishery resource, which represents the third most captured taxon (in tons) in the Brazilian Amazon River basin [23]. We implemented COI and ATP6/8 molecular analyses and used well-established algorithms for species-delimitation analyses. Our data raised a new Molecular Operational Taxonomic Units (MOTUs) within *P*. *nigricans* and certainly contributes for better estimating of biodiversity into the taxon.

## 2. Material and methods

### 2.1. Study area

**Fig 1:**
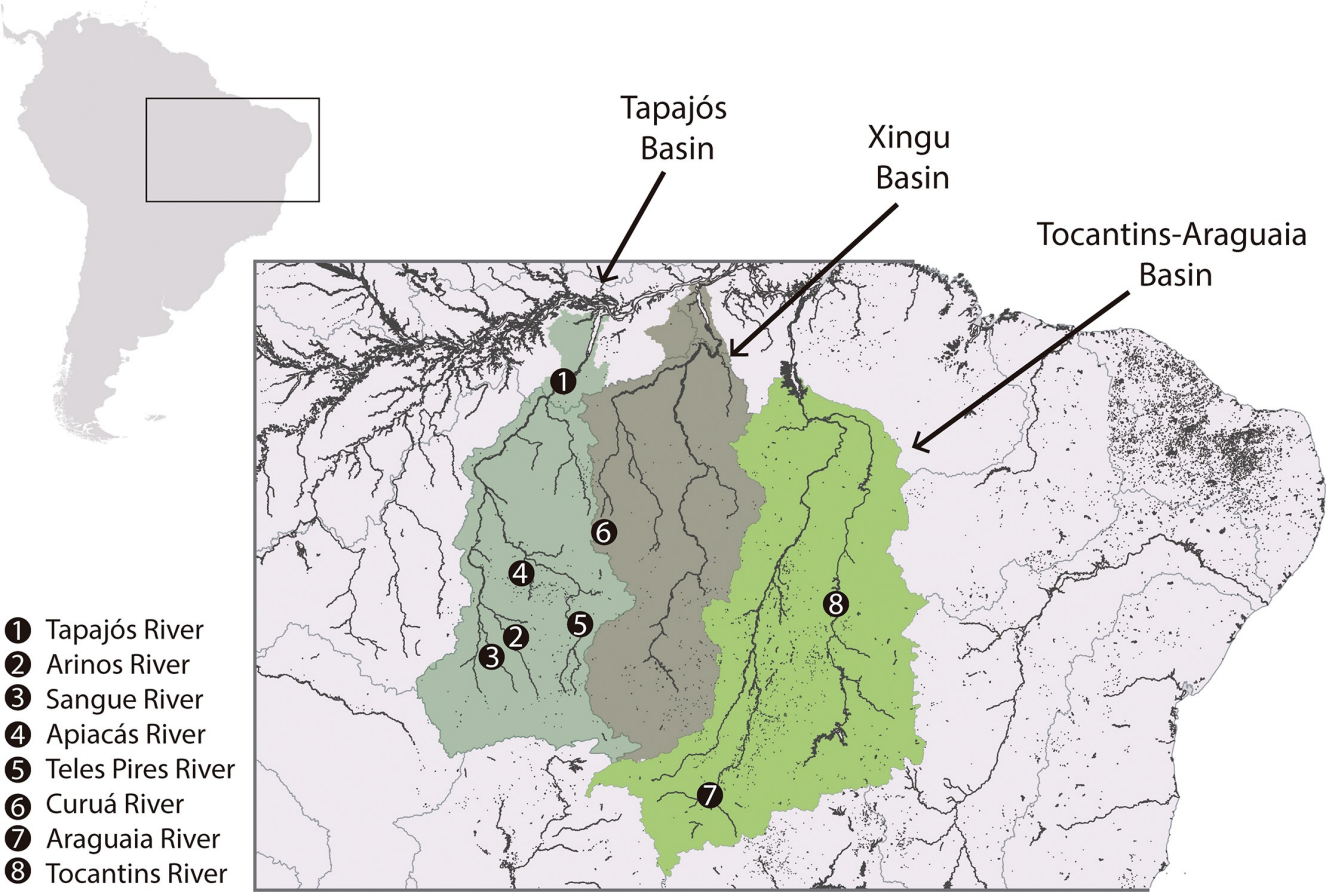
Study area. Map showing the collection sites of the *Prochilodus nigricans* samples within the Tapajós, Xingu and Tocantins-Araguaia basins. Image created via QGIS 2.18 –‘Las Palmas’ (www.qgis.org). Free vector data from Instituto Brasileiro de Geografia e Estatística—IBGE (https://www.ibge.gov.br).

The Tapajós River basin (Fig 1) is one of the largest watersheds constituting the Amazon River basin, encompassing an area of 493,986 hectares [24] and discharging approximately 6.4% of all water carried to the Amazon River [25]. This drainage hosts portion of Amazonian and Cerrado biomes, being also recognized as a peculiar ecoregion [26]. Located at the Brazilian Shield western portion, the Tapajós is a 795 km long clearwater river formed by the confluence of the Juruena and Teles Pires tributaries, whose present the length of 1240 km and 1457 km, respectively [27–29].

### 2.2. Biological sampling and ethical requirements

This study was carried out in accordance with the Brazilian law for environmental protection under the license for fish collection (SISBIO 41778–7), access of genetic material (SISGEN AAA03B9), and was approved by the Animal Ethics Committee of the Universidade Federal de São Carlos (CEUA/UFSCar 3752060715).

Biological samples of *P*. *nigricans* from the Tapajós River basin were collected during 2015. We sampled small fragments of fin tissue from adult specimens only, and most of the fish were returned alive to the river. Fin samples were additionally provided by local fishermen. We also obtained fin tissue samples from other Amazon rivers through collaborators and scientific collections (Table 1). All tissue samples were preserved in alcohol 95%, and species identification was performed based on morphological criteria, following Castro and Vari [6]. When available, new vouchers were deposited into the biological collection of the Laboratório de Ictiologia e Sistemática at Universidade Federal de São Carlos (LISDEBE/UFSCar, São Carlos, SP). Further information on this dataset, including available vouchers and Genbank (https://www.ncbi.nlm.nih.gov/nucleotide/) accession numbers are provided in Table 1.

**Table 1:** Sampling information.

ID	River	Locality, State	Accession Number	
			COI	ATP6/8
A1	Arinos^1^ *	Juara, MT	MN996677	MT052031
A2	Arinos^1^	Juara, MT	MN996681	MT052032
A3	Arinos^1^	Juara, MT	MN996682	MT052033
B1	Sangue^1^ *	Juara, MT	MN996680	MT052034
B2	Sangue^1^	Juara, MT	MN996679	MT052035
B3	Sangue^1^	Juara, MT	MN996678	MT052036
D1	Tapajós	Itaituba, PA	-	MT052043
D2	Tapajós	Itaituba, PA	MN996695	MT052051
D3	Tapajós	Itaituba, PA	MN996685	MT052041
D4	Tapajós	Itaituba, PA	MN996686	MT052042
D5	Tapajós	Itaituba, PA	MN996692	MT052040
D6	Tapajós	Itaituba, PA	MN996698	MT052053
D7	Tapajós	Itaituba, PA	-	MT052054
D8	Tapajós	Itaituba, PA	-	MT052048
D10	Tapajós	Itaituba, PA	-	MT052046
D11	Tapajós	Itaituba, PA	MN996699	MT052055
D12	Tapajós	Itaituba, PA	-	MT052050
D13	Tapajós	Itaituba, PA	-	MT052044
D14	Tapajós	Itaituba, PA	-	MT052052
D15	Tapajós	Itaituba, PA	-	MT052056
D16	Tapajós	Itaituba, PA	MN996696	MT052057
D17	Tapajós	Itaituba, PA	-	MT052058
D18	Tapajós	Itaituba, PA	-	MT052045
D20	Tapajós	Itaituba, PA	MN996701	MT052059
D21	Tapajós	Itaituba, PA	-	MT052049
D22	Tapajós	Itaituba, PA	-	MT052047
D23	Tapajós	Itaituba, PA	MN996684	-
D24	Tapajós	Itaituba, PA	MN996700	-
D26	Tapajós	Itaituba, PA	MN996697	-
D27	Tapajós	Itaituba, PA	MN996690	-
D28	Tapajós	Itaituba, PA	MN996688	-
D29	Tapajós	Itaituba, PA	MN996689	-
D30	Tapajós	Itaituba, PA	MN996691	-
D31	Tapajós	Itaituba, PA	MN996694	-
E1	Teles Pires	Sorriso, MT	MN996693	MT052037
I1	Apiacás^2^	Alta Floresta, MT	-	MT052039
I7	Apiacás^2^	Alta Floresta, MT	MN996687	-
F1	Teles Pires	Cotriguaçu, MT	MN996683	MT052038
XI01	Curuá^3^ *	Altamira, PA	MN996674	-
TA04	Araguaia	Barra do Garça, MT	MN996675	-
TA06	Araguaia	Barra do Garça, MT	MN996676	-
TA07	Araguaia	Barra do Garça, MT	MN996673	-
TA15	Araguaia	Barra do Garça, MT	MN996671	-
TA17	Araguaia	Barra do Garça, MT	MN996672	-
TA18	Araguaia	Barra do Garça, MT	MN996668	-
TA43	Tocantins	Palmas, TO	MN996670	-
TA45	Tocantins	Palmas, TO	MN996669	-
TA48	Tocantins	Palmas, TO	MN996667	-

Sample field code (ID) and collection sites of the analyzed samples of *Prochilodus nigricans* for both COI and ATP6/8 genes, and their respective Genbank accession numbers.

^1^Juruena Tributary

^2^Teles Pires Tributary

^3^Xingu Basin.

*Vouchers: A1 (LISDEBE 7257), B1 (LISDEBE 7258) and XI01 (ZMUSP 096335).

In total, we analyzed 48 samples of *P*. *nigricans*. From this total, 38 were collected through the Tapajós River basin: 28 from the Tapajós River main channel, and nine from first and second-order tributaries (six from Juruena, one from Teles Pires, and two from Apiacás rivers). Ten samples were obtained from the Xingu (1) and Tocantins-Araguaia (9) drainages (Fig 1, Table 1). Additionally, we retrieved 48 sequences of *P*. *nigricans* and some congeneric nominal species from previous studies [15,16,30–32], available at Genbank and Barcode of Life Data Base (BOLD Systems, https://www.boldsystems.org/index.php/databases) public databases (S1 Table).

### 2.3. DNA extraction, amplification, and sequencing

Total genomic DNA was extracted following the saline precipitation protocol described by Aljanabi & Martinez [33]. Each DNA sample was quantified using an Eppendorf BioPhotometer (Eppendorf, Hamburg, Germany), and standardized aliquots at 50 ng/ μL were prepared. Polymerase Chain Reaction (PCR) was carried out in order to amplify the COI and ATP synthase subunit six (ATPase6) and eight (ATPase8) genes of the mitochondrial DNA (mtDNA). The primers Fish F1 and Fish R1, and ATP 8.2_L8331 and CO3.2_H9236 were used to amplify COI and ATP6/8 regions, respectively [34,35].

Polymerase chain reactions consisted of 50 ng of DNA template, 1.25 μL of buffer (10x), 0.2 mM of dNTPs, 1 mM of MgCl2, 0.4 μM of each primer, 0,5 U Taq Platinum (Invitrogen^TM^) and ultra-pure distilled water to make up a final volume of 12.5 μL. DNA amplification reactions were carried out in an Applied Biosystems Veriti® 96-Well Thermal Cycler under the following conditions: COI—1 cycle [94°C/2 min], 35 cycles [94°C/30 sec, 59°C/30 sec, 72°C/1 min], 1 cycle [72°C/10 min], and ATP6/8, 1 cycle [94°C/3 min], 30 cycles [94°C/45 sec, 58°C/1 min, 72°C/1 min], 1 cycle [72°C/2 min]. PCR products were purified with 20% Polyethylene glycol (PEG) protocol [36] to remove unincorporated dNTPs and the excess of primers or unspecific bands. Sequencing was performed in an ABI 3730XL automatic sequencer (Applied Biosystems, Foster City, California, USA).

### 2.4. Data analyses

After sequencing, we first aligned and edited the data using ClustalW [37] and Geneious v.7.1.7 (Biomatters, Auckland, New Zealand) [38], respectively. Median Joining haplotype networks for both COI and ATP6/8 markers were reconstructed using PopART (Population Analysis with Reticulate Trees) [39]. We included 22 public sequences of *P*. *nigricans* and congeneric nominal species from the Brazilian Shield for the COI analyses. MEGA v.7 [40] was used to perform maximum-likelihood (ML) under HKY+I model and Neighbor-Joining (NJ) based on Kimura-2-parameters (K2P) analyses, both with 1000 of bootstrap replicates. Intra and intergroups genetic distances, based on K2P, with samples collected in both the Tapajós River basin and Eastern Amazon area were estimated also using the MEGA v.7 software.

General Mixed Yule Coalescent (GMYC) [41] approach was implemented for COI sequences in R with the SPLITS package (SPecies' LImits by Threshold Statistics) [42], considering the single threshold under the default settings (interval = c(1,10)). GMYC combines stochastic lineage growth models with coalescence ones to detect intra and interspecific evolutionary processes (coalescence and speciation/extinction events, respectively) and has been commonly applied to identify MOTUs in studies concerning taxonomic issues [43–46]. For this analysis, a COI ultrametric topology was produced using the Bayesian Inference (BI) method implemented by BEAST v.2.0 [47] in CIPRES Science Gateway [48] (www.phylo.org). We also used the site model, based on Bayesian Information Criteria (BIC), as suggested by JModeltest [49] (COI = HKY+I), and lognormal relaxed molecular clock model and birth-death tree prior, according to Costa-Silva *et al*. [50]. Two independent runs of four Markov chains of 120 million generations were conducted, sampling every 10000 steps, with 30% of the first topologies being discarded as burn-in. The combination of the independent tree and log files was performed with the LogCombiner v.1.8 software [51] and stationarity and convergence were assessed with Tracer v1.5 [52], considering values of the effective sample size (ESS) of all parameters equal or higher than 200. A maximum clade credibility tree was summarized in TreeAnnotator v.1.8 [53], and later visualized in the FigTree v.1.4 software, which is available at http://tree.bio.ed.ac.uk/software/figtree/.

GMYC, ML, and NJ analyses were performed gathering all COI dataset, which comprised the newly generated sequences in addition to those downloaded from the databases.

## 3. Results and discussion

The total dataset generated in this study consisted of 35 COI and 29 ATP6/8 sequences, after alignment and editing (Table 1). COI fragments ranged from 561 to 564 bp and presented 26 polymorphic sites and 16 parsimony informative ones. The average ATP6/8 sequence length was 985 bp and included 18 variable sites, 11 of which were parsimony informative. The COI and ATP6/8 networks included 19 and 12 haplotypes, respectively, revealing individuals within the Tapajós mainstream assigned to highly divergent haplotypes, showing at least nine and six mutational steps from the most frequent haplotype for COI and ATP6/8, respectively (See S1 Fig).

**Fig 2:**
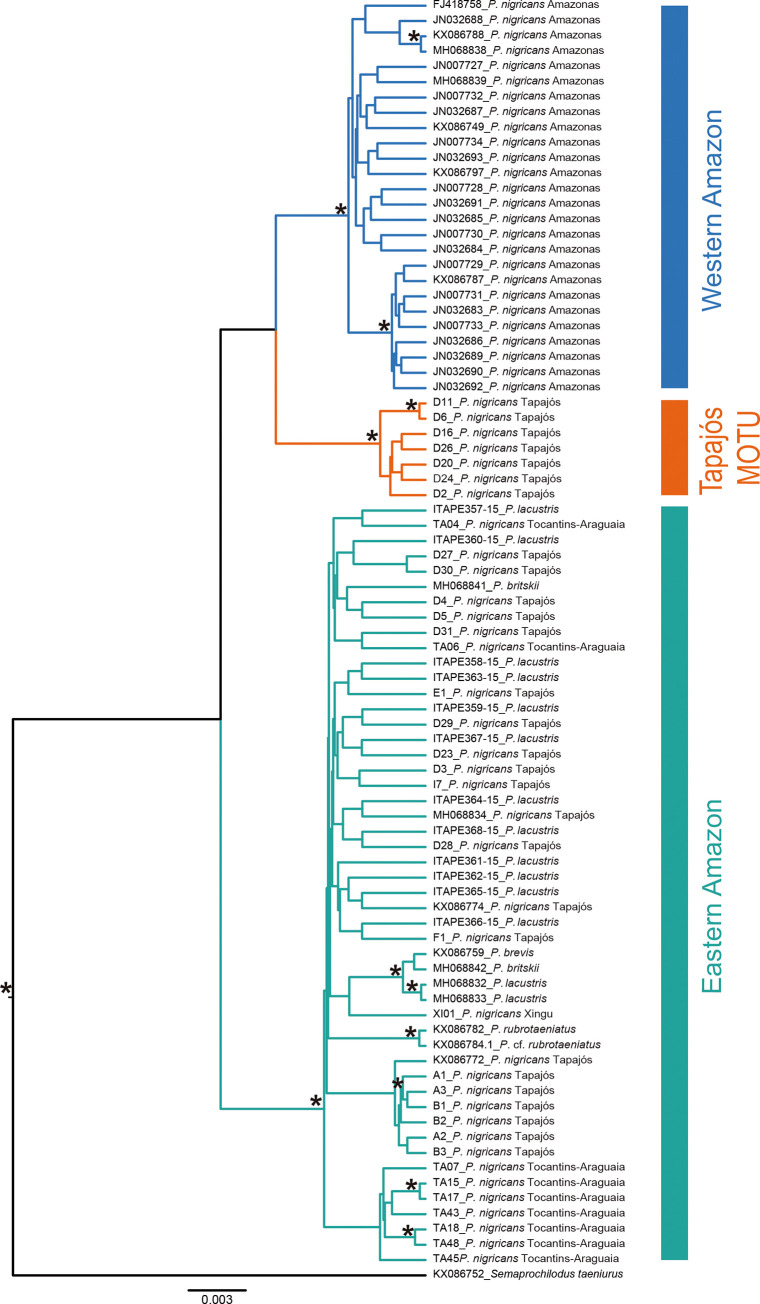
Bayesian Inference used to delimitate *Prochilodus nigricans* lineages within the Amazon River basin using GMYC analysis. Blue and green clades represent the Western and Eastern Amazon previously defined clusters, respectively. Orange clade represents the newly detected lineage (Tapajós MOTU). Posterior probability values (>0.94) are represented by asterisks above nodes.

The maximum likelihood results of the GMYC model performed with COI was significantly higher (L = 696.0997) than that of the null model (L0 = 642.3439), allowing us to reject the hypothesis that all individuals belong to the same molecular unit. The GMYC single threshold analysis revealed the occurrence of three MOTUs within the Amazon River basin (Fig 2), increasing the number of MOTUs reported in previous studies [15,16] in this large basin. Our study recovered the previous Western and Eastern Amazon MOTUs (showed, respectively, in blue and green clusters in Fig 2) and raised an additional one, named hereafter Tapajós MOTU (orange cluster in Fig 2).

The pairwise K2P distances between this new molecular lineage and rest of the Eastern group were 0.017 ± 0.004 and 0.01 ± 0.003 for COI and ATP6/8, respectively. Although both values were lower than that commonly used as an initial threshold for the molecular identification approach [54], our distance values were similar to those found between other *Prochilodus* species, which ranged from 1.2% to 10.3% [16]. The distances within groups using the COI data were 0.011 ± 0.002 for the Eastern group and 0.002 ± 0.001 for the new MOTU, while with ATP6/8 it ranged from 0.001 ± 0.000 and 0.002 ± 0.001 for the Eastern group and the new MOTU, respectively.

It is noteworthy that two of the *P*. *nigricans* MOTUs observed here are co-occurrents into the Tapajós River, and in the phylogenetic tree were shown as paraphyletic (Fig 2). This result corroborates previous biogeographic findings, in which the Tapajós River basin was seen as non-monophyletic, indicating a possible hybrid origin of this hydrographic system [17]. As stated by Dagosta and de Pinna [17], the Tapajós mainstream is related to the rivers of the Western Amazon, while the Juruena and Teles Pires tributaries are related to the Eastern drainages of the Brazilian shield. The same pattern of relationship was here verified among the *P*. *nigricans* lineages through the GMYC analysis (Fig 2), which was also well supported by the ML (>79) and NJ (>74) topologies (S2 Fig). The Tapajós and Western Amazon lineages come up as sister groups, whereas the samples of *P*. *nigricans* collected within the tributaries of the Tapajós River (See Table 1 and S1 Table) represented the Eastern Amazon lineage. It is worthy to note that individuals of both Tapajós and Eastern Amazon lineages were seen in sympatry at least in part of the collection sites. Given the entanglement of the Amazon River basin, diverse phylogeographic hypotheses [55,56] have been hitherto postulated to explain it. Within this framework, and considering our results, *P*. *nigricans* raises a possible and interesting model to test such ideas. Different migratory fish groups such as *Brycon* [57], *Leporinus* [5], and *Zungaro* [19] have been showing hidden diversity, particularly in the Tapajós River basin. Overall, these results appear indicating both the high level of endemism [18] and the putative historical hybridism [6] can be important drives of fish diversity in this peculiar system.

Our findings reinforce the importance of molecular species delimitation approach in investigating hidden biodiversity into the Amazon ichthyofauna. Since the Amazon River basin is in the spotlight as a candidate area for the construction of diverse hydroelectric plants [58,59] and threatened by other factors, such as overharvesting, deforestation and climate change [13,60,61], the development of studies focusing on biodiversity survey is of paramount importance for effective conservation management plans [3,62,63], aiming at maintaining the maximum genetic diversity and evolutionary potential for a species [64,65].

Overall, the present study was able to reveal hidden biodiversity in *P*. *nigricans*, delimitating genetic lineages, and helping to characterize a new MOTU that must be better investigated to confirm the existence of a new species. Moreover, our findings raise new insights for a further approach related to the possible historical hybrid origin of the Tapajós River basin.

## Supporting information

S1 Fig Haplotype networks using samples from the Tapajós River basin and the Eastern Amazon clade. A. COI, B. ATP6/8.
(TIF)

S2 Fig COI topologies including the generated data and the sequences retrieved from public databases. A. Maximum likelihood, B. Neighbor joining.
(TIF)

S1 Table (DOCX)
