# Supplementary figures and images for: Hidden diversity in Prochilodus nigricans: A new genetic lineage within the Tapajós River basin
Source: PLoS One. 2020 Aug 25;15(8):e0237916. doi: 10.1371/journal.pone.0237916 (PMC7447553; doi:10.1371/journal.pone.0237916)

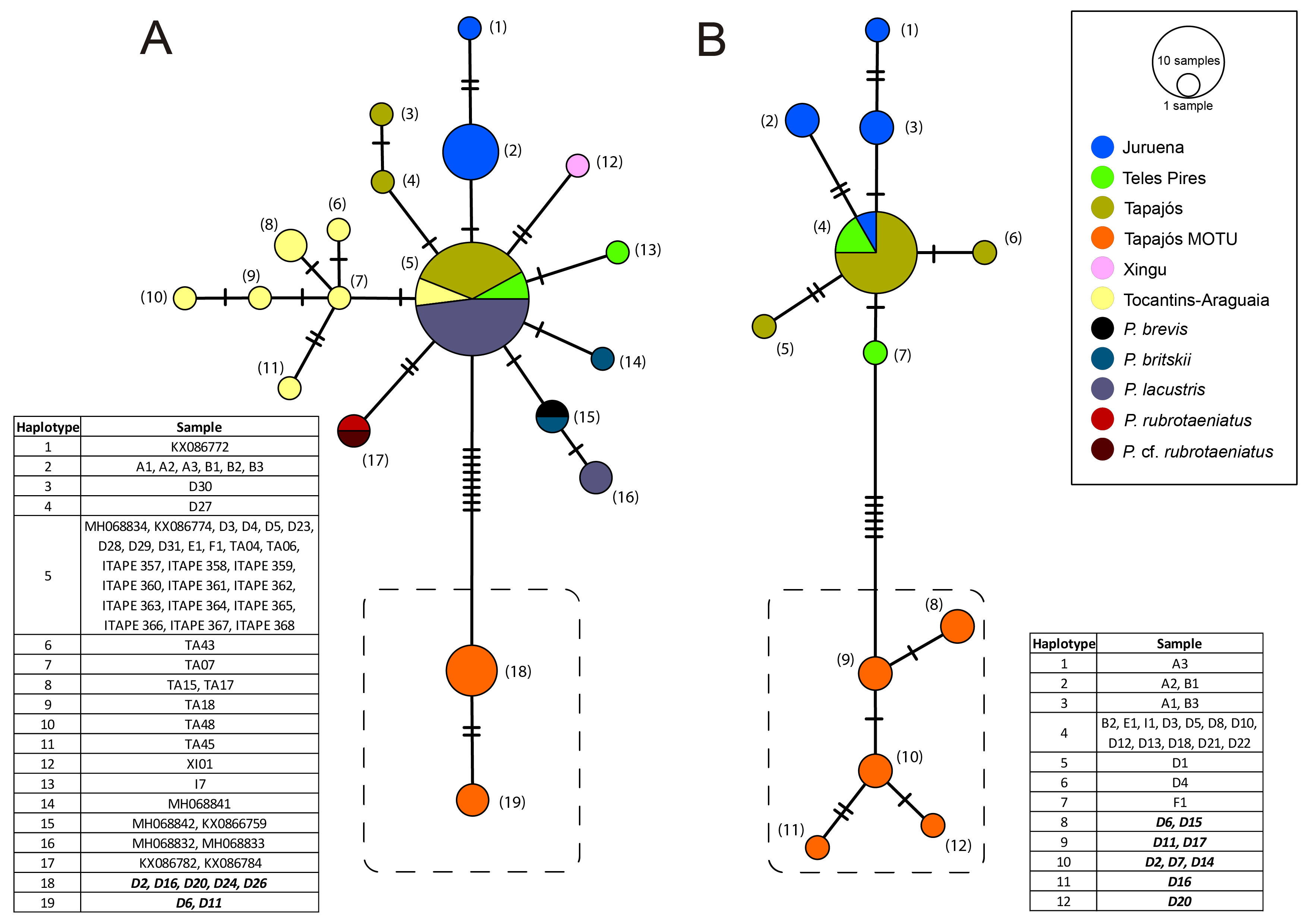

Supplement: S1 Fig — A. COI, B. ATP6/8. (TIF) [file pone.0237916.s001.tif]

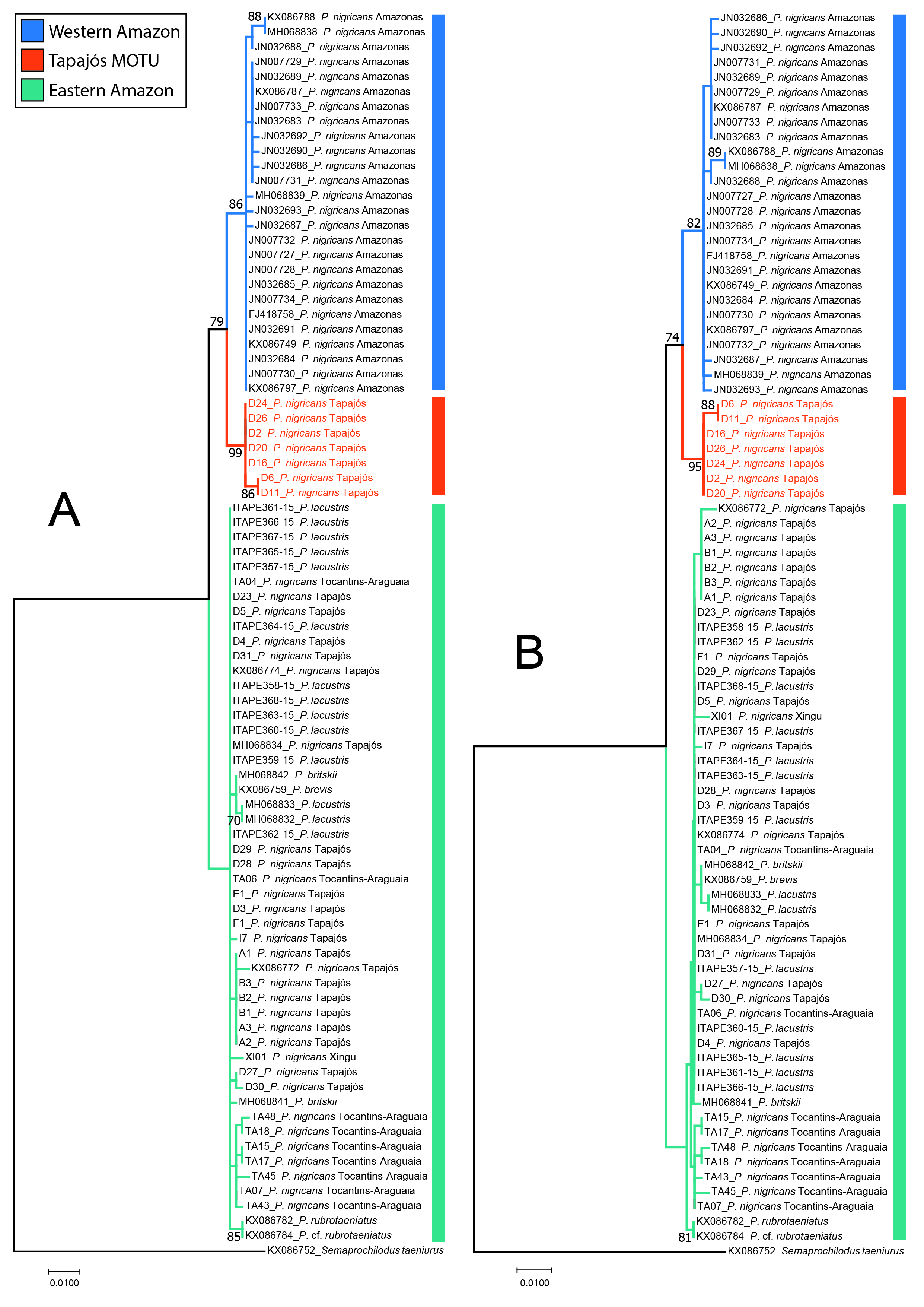

Supplement: S2 Fig — A. Maximum likelihood, B. Neighbor joining. (TIF) [file pone.0237916.s002.tif]
