# Supplementary material for: Hidden diversity in Prochilodus nigricans: A new genetic lineage within the Tapajós River basin
Source: PLoS One. 2020 Aug 25;15(8):e0237916. doi: 10.1371/journal.pone.0237916 (PMC7447553; doi:10.1371/journal.pone.0237916)
Supplement: S1 Table — (DOCX) [file pone.0237916.s003.docx]

**S1 Table.**

| **Species** | **Code** | **River** | **Sampling site** | **Ac. number** | **Source** |
| --- | --- | --- | --- | --- | --- |
| *P. nigricans* | M3-7 | Amazonas | Manaus, AM | FJ418758 | Ardura *et al.* 2010 |
| *P. nigricans* | Curimata1 | Amazonas | Manaus, AM | JN007727 | Ardura *et al.* 2013 |
| *P. nigricans* | Curimata4 | Amazonas | Manaus, AM | JN007728 | Ardura *et al.* 2013 |
| *P. nigricans* | Curimata5 | Amazonas | Manaus, AM | JN007729 | Ardura *et al.* 2013 |
| *P. nigricans* | Curimata6 | Amazonas | Manaus, AM | JN007730 | Ardura *et al.* 2013 |
| *P. nigricans* | Curimata7 | Amazonas | Manaus, AM | JN007731 | Ardura *et al.* 2013 |
| *P. nigricans* | Curimata8 | Amazonas | Manaus, AM | JN007732 | Ardura *et al.* 2013 |
| *P. nigricans* | Curimata9 | Amazonas | Manaus, AM | JN007733 | Ardura *et al.* 2013 |
| *P. nigricans* | Curimata10 | Amazonas | Manaus, AM | JN007734 | Ardura *et al.* 2013 |
| *P. nigricans* | H1 | Amazonas | Manaus, AM | JN032683 | Ardura *et al.* 2013 |
| *P. nigricans* | H2 | Amazonas | Manaus, AM | JN032684 | Ardura *et al.* 2013 |
| *P. nigricans* | H3 | Amazonas | Manaus, AM | JN032685 | Ardura *et al.* 2013 |
| *P. nigricans* | H4 | Amazonas | Manaus, AM | JN032686 | Ardura *et al.* 2013 |
| *P. nigricans* | H5 | Amazonas | Manaus, AM | JN032687 | Ardura *et al.* 2013 |
| *P. nigricans* | H6 | Amazonas | Manaus, AM | JN032688 | Ardura *et al.* 2013 |
| *P. nigricans* | H7 | Amazonas | Manaus, AM | JN032689 | Ardura *et al.* 2013 |
| *P. nigricans* | H8 | Amazonas | Manaus, AM | JN032690 | Ardura *et al.* 2013 |
| *P. nigricans* | H9 | Amazonas | Manaus, AM | JN032691 | Ardura *et al.* 2013 |
| *P. nigricans* | H10 | Amazonas | Manaus, AM | JN032692 | Ardura *et al.* 2013 |
| *P. nigricans* | H11 | Amazonas | Manaus, AM | JN032693 | Ardura *et al.* 2013 |
| *P. nigricans* | LBP 1690 | Amazonas | Manaus, AM | KX086749 | Melo *et al.* 2016 |
| *P. nigricans* | LBP 8589 | Arinos/Tapajós | Diamantino, MT | KX086772 | Melo *et al.* 2016 |
| *P. nigricans* | LBP 12865 | Tapajós | Itaituba, PA | KX086774 | Melo *et al.* 2016 |
| *P. nigricans* | OS 18792 | Itaya/Amazonas | Maynas, Loreto | KX086787 | Melo *et al.* 2016 |
| *P. nigricans* | OS 18792 | Itaya/Amazonas | Maynas, Loreto | KX086788 | Melo *et al.* 2016 |
| *P. nigricans* | FMNH 113534 | Itaya/Amazonas | Iquitos, Loreto | KX086797 | Melo *et al.* 2016 |
| *P. nigricans* | LBP 12865 | Tapajós | Itaituba, PA | MH068834 | Melo *et al.* 2018 |
| *P. nigricans* | LBP 174 | Acre/Purus | Rio Branco, AC | MH068838 | Melo *et al.* 2018 |
| *P. nigricans* | LBP 10919 | Madeira | Porto Velho, RO | MH068839 | Melo *et al.* 2018 |
| *P. brevis* | LBP 2496 | Açude Araçá, | Macaíba, RN | KX086759 | Melo *et al.* 2016 |
| *P. britskii* | LBP 20260 | Apiacás | Alta Floresta, MT | MH068841 | Melo *et al.* 2018 |
| *P. britskii* | LBP 19615 | Apiacás | Alta Floresta, MT | MH068842 | Melo *et al.* 2018 |
| *P. lacustris* | LBP 9104/42735 | Poti | Teresina, PI | MH068833 | Melo *et al.* 2018 |
| *P. lacustris* | LBP 9104/42733 | Poti | Teresina, PI | MH068832 | Melo *et al.* 2018 |
| *P. lacustris* | UEMA 104603 | Itapecuru | Rosário, MA | ITAPE357-15* | Melo *et al.* 2018 |
| *P. lacustris* | UEMA 104603 | Itapecuru | Rosário, MA | ITAPE358-15* | Melo *et al.* 2018 |
| *P. lacustris* | UEMA 104603 | Itapecuru | Rosário, MA | ITAPE359-15* | Melo *et al.* 2018 |
| *P. lacustris* | UEMA 104603 | Itapecuru | Rosário, MA | ITAPE360-15* | Melo *et al.* 2018 |
| *P. lacustris* | UEMA 104603 | Itapecuru | Rosário, MA | ITAPE361-15* | Melo *et al.* 2018 |
| *P. lacustris* | UEMA 104603 | Itapecuru | Rosário, MA | ITAPE362-15* | Melo *et al.* 2018 |
| *P. lacustris* | UEMA 104603 | Itapecuru | Rosário, MA | ITAPE363-15* | Melo *et al.* 2018 |
| *P. lacustris* | UEMA 104603 | Itapecuru | Rosário, MA | ITAPE364-15* | Melo *et al.* 2018 |
| *P. lacustris* | UEMA 104603 | Itapecuru | Rosário, MA | ITAPE365-15* | Melo *et al.* 2018 |
| *P. lacustris* | UEMA 104603 | Itapecuru | Rosário, MA | ITAPE366-15* | Melo *et al.* 2018 |
| *P. lacustris* | UEMA 104603 | Itapecuru | Rosário, MA | ITAPE367-15* | Melo *et al.* 2018 |
| *P. lacustris* | UEMA 104603 | Itapecuru | Rosário, MA | ITAPE368-15* | Melo *et al.* 2018 |
| *P.* cf*. rubrotaeniatus* | ANSP 40692 | Orinoco | La Esmeralda, Amazonas | KX086784 | Melo *et al.* 2018 |
| *P. rubrotaeniatus* | USNM 403693 | Essequibo | Cuyuni-Mazaruni | KX086782 | Melo *et al.* 2018 |
| *Semaprochilodus taeniurus* | LBP 1691 12759 | Amazonas | Manaus, AM | KX086752 | Melo *et al.* 2018 |

* Sequences retrieved from BOLD Systems.
